# Bilateral claw hand deformity: a rare image

**DOI:** 10.11604/pamj.2024.48.26.43370

**Published:** 2024-05-29

**Authors:** Angan Ghosh, Sanjot Ninave

**Affiliations:** 1 Department of Anesthesia, Jawaharlal Nehru Medical College, Datta Meghe Institute of Higher Education and Research, Sawangi, India

**Keywords:** Ulnar nerve damage, Hansen’s disease, Zancolli lasso procedure

## Image in medicine

A 58-year-old male came to the hospital with the complaint of difficulty in opening both hands and inability to perform routine activities. On inspection, fingers were bent with atrophy of the intrinsic muscles of both hands. On routine evaluation, it was noted that the patient had a past history of Hansen´s disease for which he took multidrug therapy (MDT) consisting of rifampicin, dapsone, and clofazimine for 6 months. The patient was then examined thoroughly to check the strength and flexibility of the fingers and an electromyography test was done. A diagnosis of bilateral claw hand was made due to ulnar nerve damage. The patient was advised physical therapy along with strengthening exercises to gain more flexibility in fingers and braces to prevent further nerve injury. The patient was posted for a Zancolli lasso procedure in the following hospital visit. Routine investigations and radiological imaging were normal and the surgery was done under a brachial plexus block with tourniquet control using local anaesthetics. Intraoperatively the patient was vitally and hemodynamically stable. The surgery was uneventful and postoperative rehabilitation was done.

**Figure 1:**
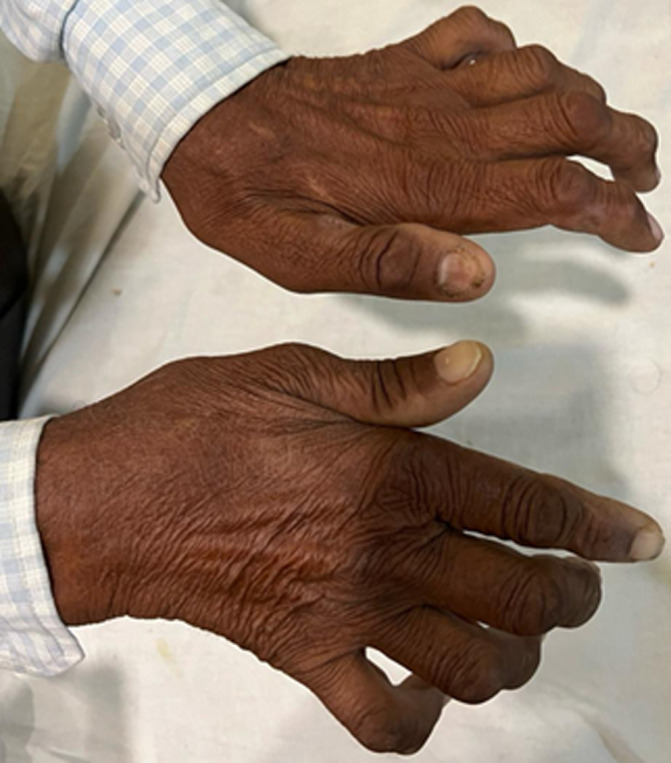
a 58-year-old male with bilateral claw hand deformity

